# Supplementary material for: Are carbon emissions trading and green financial instruments synergistic? -Comprehensive quantitative research based on content analysis
Source: PLoS One. 2024 Mar 7;19(3):e0298601. doi: 10.1371/journal.pone.0298601 (PMC10919601; doi:10.1371/journal.pone.0298601)
Supplement: S1 Data — (ZIP) [file pone.0298601.s001.zip › Data/地级市Z_F工作报告.zip!/║■▒▒/╢⌡╓▌╩╨/└┤╫╘╣1⁄2╓┌║┼í╛┬φ┐╦╩2╛▌═°í┐_2012.docx]

各位代表：

　　现在，我代表市人民政府向大会报告本届政府过去四年的工作，对未来五年和今年的工作提出建议，请予审议，并请各位市政协委员和列席人员提出意见。

　　一、过去四年工作的回顾

　　过去四年是极不平凡的四年。四年来，在省委、省政府和市委的正确领导下，全市上下深入贯彻落实科学发展观，抢抓重大发展机遇，积极应对国际金融危机和各种自然灾害的严重冲击，全面完成了市六届人大一次会议确定的各项任务，经济和社会发展取得巨大成就。

　　——综合实力大幅提升。全市生产总值、财政总收入、地方一般预算收入四年翻一番，2011年分别达到465亿元（预计，下同）、41.3亿元和25.9亿元，年均增长分别达到15.5%、18.5%和32.1%。

　　——产业结构不断优化。工业主体地位显著增强，工业增加值三年翻一番，工业化率达到52.6%，居全省第一。钢铁深加工、金刚石刀具、生物医药等成为省重点成长型产业集群。三次产业结构由2007年的14.6：51.2：34.2调整为13：57.9：29.1。

　　——改革开放取得突破。重点领域和关键环节改革不断深化，共承担了36项国家和省级改革试点任务。推行大部门制改革，市级政府部门由32个精简为27个。深化行政审批制度改革，审批事项由470项审减到224项。城乡一体化试点、综合改革示范稳步推进。开放型经济快速发展，四年累计外贸出口4.8亿美元，年均增长22.6%。

　　——城市功能日益完善。按照“全域鄂州”理念，编制了城乡总体规划，建立了“一主三新十特百新社区”空间布局体系。2011年主城区完成基础设施投资7.21亿元，四年累计完成城镇基础设施投资50亿元，全市新增建成区面积33平方公里，主城区建成区面积达到50.6平方公里，城镇化率达到58%，居全省第二。

　　——社会事业全面发展。就业形势稳中向好，城镇登记失业率四年均控制在4.3%以内，城乡一体的社会保障体系逐步健全。新建了吴都中学、城市福利中心、三国吴都博物馆，素质教育整体推进，科技、文化事业快速发展，群众看病难、看病贵不断缓解，社会保持和谐稳定。

　　——人民生活明显改善。2011年，城镇居民人均可支配收入达到16800元，农村居民人均纯收入达到7640元，年均分别增长11.6%和14.8%。城乡居民收入差距从2007年的2.46:1缩小到2.2:1。

　　四年来，鄂州获得国家园林城、全国科技进步先进城市、全国法制宣传教育先进城市、国家“数字城市”建设示范市、中部最佳投资城市、全国文明城市提名资格、中国人居环境范例奖等荣誉称号。我们围绕加快发展主要做了以下工作：

　　（一）坚持扩大发展规模不放松。着力扩大投资。始终把投资作为推动经济增长的第一拉动力，出台了《关于扩大投资促进经济平稳较快增长的若干意见》，连续四年实施“三个一百”工程，落实重点项目四大家领导挂点、派驻工作队和市直部门跟踪服务等制度。2011年完成固定资产投资336亿元，增长30%。四年完成固定资产投资1005.5亿元，年均增长38.2%；实施亿元以上项目405个，一批重大项目建成投产。大力招商引资。出台了《关于加快发展开放型经济的若干意见》和《关于深入推进招商引资工作的实施意见》，坚持市领导带队招商与小分队招商结合、走出去与请进来并重。2011年招商引资180亿元，增长36.4%。四年招商引资462.9亿元，实际利用外资4.24亿美元，年均分别增长42.5%和11.2%。全力争取中省投资。2011年争取中省投资项目189个，到位资金8.3亿元。四年争取中省投资项目848个，总投资19.5亿元。加快发展区街经济。实施扩权强区27条政策措施，鄂城区连续三年进入全省县域经济二十强，华容区排在第二方阵，梁子湖区进位明显，街道经济快速发展，区街财政总收入占全市的比例由40%提高到58.3%。

　　（二）坚持构建发展支撑不放松。加快发展工业经济。实施工业强市战略，一手抓壮大规模，一手抓调整结构，制定了八大产业振兴和发展三年行动方案，实施了工业双千亿元产业发展计划，建立了重点企业周监测、旬分析、月调度制度和“一企一策”扶持计划，工业竞争力显著提升。2011年规模以上工业增加值260亿元，年均增长25.1%。一批核心竞争力强的规模企业脱颖而出，规模以上、销售收入过亿元工业企业达到540家、120家，分别比2007年增加238家、84家。高新技术产业增加值64.4亿元，年均增长34.4%。多佳、枫树、鄂州武昌鱼等获中国驰名商标，顾地获中国名牌，多佳、方方等8件商标获准了商标国际注册，鄂钢、鄂重、四海湖、信源等25件商标被认定为湖北省著名商标。加快发展现代服务业。实施《鄂州市服务业跨越发展规划》、《关于加快发展旅游业的若干意见》。2011年社会消费品零售总额151亿元，年均增长15.6%；服务业增加值137.9亿元，年均增长15.8%。支持金融业发展，市财政每年安排1000万元用于企业技改项目贷款贴息，安排100万元奖励金融机构支持企业发展，汉口银行、中信银行等相继进驻鄂州，农村信用社完成法人改革。全市金融机构各项存贷款余额四年增长超过1.3倍。连续四年被评为“A级信用城市”。切实转变发展方式。全市整体纳入“青阳鄂”大循环经济示范区，大梁子湖“两型”社会示范区上升为省级战略，建设了鄂钢生产工艺循环等项目，实施了鄂钢TRT发电、球团厂烟尘治理等重点工程，节能减排目标全面实现。

　　（三）坚持拓展发展平台不放松。全力加快城市基础设施建设。确立了宜居宜业组群式大城市的发展目标，连续四年加大城市基础设施建设，新建了鄂州大道、吴都大道、吴楚大道中段等道路工程，完成了武昌大道、江碧路等21条道路刷黑工程，实施了三国吴都风光带一、二期和洋澜湖生态公园等绿化景观工程，连续四年投资1亿余元改造背街小巷120条。电网累计投资9.47亿元，110千伏容载比居全省第一。建成了城区垃圾处理场、污水处理厂和50座公厕，实施了“城管执法进街道”，城市管理重心下移，拆除违法建设22.3万平方米，规范设置市容疏导场所，城市功能不断提升，人居环境明显改善。超常规发展交通事业。完成了樊口大桥、电排站桥、汽李线、葛湖线等干线建设，汉鄂高速、鄂黄高速鄂州段、火车站改造等项目加快推进。武汉新港鄂州港区建设进展顺利，三江港区被列入武汉新港核心港区，三和管桩综合码头等14个深水码头加快建设。2011年完成交通建设投资15.6亿元，四年完成投资40.6亿元，公路密度居全省第一，基本形成鄂东综合交通枢纽架构。不断加大开发区建设力度。葛店开发区共投入10亿元，实施了建设大道、东湖路、高新东西路、污水处理厂等工程。鄂州开发区共投入8亿元，实施了旭光大道、樊川大道、污水处理厂等项目。花湖工贸新城、红莲湖旅游新城、梧桐湖新区等承载能力不断增强。

　　（四）坚持激活发展动力不放松。以改革促发展，城乡一体化试点和综合改革示范上升为省级战略。加快推进城乡一体化。积极探索了工农一体、城乡互促、基础设施共建共享的城乡一体化发展模式。成功承办全省城乡一体化工作会议。积极推进特色镇建设，市区镇三级每年筹资6000万元开展特色镇建设。大力建设农村新社区，全面推进城乡公共服务均等化，探索出“1+8”农村新社区服务新平台。现代都市农业加快发展，水产、畜禽、蔬菜、林果等四大支柱产业增加值占农业增加值的71.9%。推进农业产业化，省、市级龙头企业分别达到7家和47家，农业产业化经营组织648家。华容农产品加工园被列为全省重点农产品加工园。加强农产品品牌建设，认证无公害农产品基地38个、无公害产品46个、绿色食品30个。改善农村生产生活条件，建成通村公路926公里，在全省率先实现了村村通达水泥路；巩固和改善了防洪、灌溉工程体系；完成土地整理47万亩,我市被列入国家级土地整治示范区。农业综合机械化水平达63%。自来水主管网实现乡镇全覆盖，65万农村居民喝上自来水。筹资1亿元开展农村环境综合连片整治，农村人居环境明显改善。集体林权制度、农村产权制度等改革稳步推进。完成了第二轮30个重点村整村推进扶贫计划。努力推进综合改革示范工作。高标准做好顶层设计，编制的《规划纲要》和《总体方案》得到省政府批复。成功承办了全省推进综合改革示范工作现场会，出台了《关于加快体制机制创新推进综合改革示范工作的实施意见》，集中优势力量，推进综合改革。突出核心示范，确立了五大领域32项改革，将全市划分为绿色发展区、创新发展区、转型发展区三大板块，整合设立五大功能区，对五大功能区内的200平方公里基础设施建设进行了总体规划。策划实施了综改示范26个启动项目，总投资366.01亿元。积极争取省直部门支持，主动开展项目对接，吴楚大道、梁子湖水生态展示馆等21个项目前期工作进展顺利，完成投资50.2亿元。

　　（五）坚持共享发展成果不放松。把保障和改善民生作为政府工作的出发点和落脚点。努力办好“十件实事”。2011年“十件实事”全面完成。四年投入办实事资金36.07亿元，百万市民受惠。努力提高人民生活水平。创建省级创业型城市，支持全民创业，以创业带动就业，四年发放小额担保贷款2.3亿元，新增个体私营经济市场主体24865户，带动城镇新增就业8万人，下岗失业人员再就业2.5万人。提高了最低工资标准和企业退休人员养老金。在全省率先实现医疗保险城乡一体化和养老保险城乡全覆盖，城镇职工医保、城镇居民医保和新农合实现“三网合一”，全市医疗保险综合参保率达到98.5%，城乡居民养老保险参保率达到90%。投资1.5亿元，建成城乡养老机构26个，城乡养老服务体系基本形成；城乡低保实现“应保尽保”。大力实施保障性安居工程，四年新建廉租住房3700套、经济适用住房200套、公共租赁住房1750套，发放住房租赁补贴1220万元。四年发放住房公积金个人贷款9.28亿元，6648户中低收入家庭圆了住房梦。努力发展社会事业。加快发展教育事业，全面实施中小学校舍安全工程，新改扩建校舍97栋。实施农村中小学布局调整，建成40所农村寄宿制学校。武汉城市圈（鄂州）职教园获省政府批准建设，已有4所院校入驻。鄂州职业大学跻身全国高职院校200强。加快发展科技事业，实施中省科技计划项目122项，获省级科技进步奖18项，新增专利申请2630件。全面启动医药卫生体制改革，积极开展全国公立医院改革试点，在基层医疗卫生机构全面推行基本药物制度全覆盖，药价下降48%。投资2亿元，加强了市直医院和基层医疗机构设施建设，公共卫生均等化服务实现了全覆盖。发展文体广电事业，在全省率先实现综合文化站、农民体育健身工程、农家书屋全覆盖，实施城乡电影“2131”工程，率先启动农村有线电视“三网融合”。“周周乐”广场文化活动入选湖北省15大宣传文化活动品牌。鄂州雕花剪纸和牌子锣分别入选联合国和国家级非物质文化遗产名录。鄂州籍运动员首次在亚运会、世界大学生运动会上勇夺金牌。人才工作取得显著成效。低生育水平进一步稳定，基层基础工作进一步加强，出生人口性别比升高趋势得到遏制。社区建设和村民自治工作深入开展。国防教育和国防后备力量建设不断加强，军政军民团结更加巩固。“五五普法”全面完成，“六五普法”开局良好。全面提升应急管理水平，各类突发事件得到妥善处置。大力整顿市场秩序，食品药品监管进一步加强。排查治理重点行业和重点领域的安全隐患，安全生产保持平稳态势。开展全省社会管理创新试点，连续四年实现命案全破，社会保持和谐稳定。圆满完成了四川省汉源县九襄镇灾后重建对口援建任务和支援新疆精河县工作，援建新疆农五师八十四团和恩施州咸丰县的工作顺利推进。

　　（六）坚持优化发展环境不放松。以建设法治政府为目标，扎实开展科学发展观学习实践、创先争优、执行力建设和治庸问责等活动，政府的宗旨意识和公务员能力建设得到加强。推进法治政府建设。加快构建“职权法定、流程再造、绩效考评、行政问责”为主要架构的依法行政体系。认真执行市人大及其常委会决议，广泛听取市政协和各民主党派意见，自觉接受人大的法定监督、政协的民主监督和新闻舆论监督，认真办复人大代表和政协委员议案、提案和建议，四年办理1592件，满意率和基本满意率不断提高。优化行政服务环境。不断加强政府执行力建设，四级行政服务体系实现全覆盖，全面推行《鄂州市市直机关公务员通用能力席位标准（试行）》，政务环境进一步优化。构建廉政风险防控体系。按照国家廉政风险预警防控工作试点和全省廉政风险预警防控工作示范市的要求，构建廉政风险教育、排查、化解、防范机制。严格规范土地出让、政府采购、国有资产管理，强化政府投资项目审计，实施行政问责和效能监察，增强政府工作人员廉洁从政意识，政府形象有效提升。

　　与此同时，社会科学、文学艺术、档案方志、新闻出版、民族宗教、外事侨务、人民防空、统计、物价、对台、保密、粮食、供销、工会、青年、妇女、儿童、老龄、侨联、残联、机关事务、气象、邮政、通讯、无线电、石油石化、烟草等工作都取得了新的成绩。

　　各位代表，四年的成就令人振奋，积累的经验弥足珍贵。在工作中，我们深切感受到：必须坚持解放思想，不断深化对鄂州市情的再认识，积极探索符合鄂州实际的特色发展之路；必须坚持发展至上，紧紧扭住经济建设这个中心，创造性地开展工作，不断增强发展的动力和活力；必须坚持夯实基础，始终注重抓当前与谋长远的有机结合，拓展发展空间，增强发展后劲；必须坚持以人为本，切实保障和改善民生。

　　各位代表，四年来鄂州经济和社会发展的成绩是在极其复杂严峻的挑战中取得的，成果来之不易。我代表市人民政府向辛勤工作在各个岗位上的广大干部群众，向各位人大代表、政协委员，向各民主党派、工商联、无党派人士、人民团体和社会各界人士，向为鄂州发展做出贡献的各位老同志，向解放军指战员、武警官兵以及所有来鄂州的投资者、劳动者和海内外朋友表示崇高的敬意和衷心的感谢！

　　四年的发展成就令人鼓舞，但我们必须清醒地看到面临的矛盾和问题。主要是：经济规模不大、产业结构不优、整体抗风险能力不强；制约经济社会发展的一些深层次矛盾还未根本消除，深化改革的任务还十分艰巨；一些关系人民群众切身利益的问题还需继续下大力气解决；经济发展的环境还有待进一步改善，政府自身改革和建设有待进一步加强，等等。我们必须认真面对，切实加以解决。

　　二、今后五年工作的建议

　　各位代表，今后五年，是鄂州全面推进综合改革示范工作的攻坚时期，也是鄂州科学发展、跨越发展的重要战略机遇期，更是鄂州加快建设宜居宜业组群式大城市的关键时期。国家“十二五”规划的实施、全省“两圈一带”战略的推进，为我们带来了前所未有的机遇。国际金融危机的持续影响、世界经济发展的不确定性、我市经济发展的结构性矛盾，使我们面临严峻的挑战。省委、省政府对鄂州改革发展寄予厚望，我们一定要积极抢抓机遇，有效应对挑战，争创一流业绩，推进跨越发展。

　　今后五年政府工作的总体要求是：高举中国特色社会主义伟大旗帜，以邓小平理论和“三个代表”重要思想为指导，深入贯彻落实科学发展观，抢抓省委省政府加快实施“两圈一带”重大战略机遇，以科学发展、跨越发展为主题，以加快转变经济发展方式为主线，以加速新型工业化、新型城镇化和农业现代化进程为重点，围绕建设产业鄂州、创新鄂州、全域鄂州、灵秀鄂州、幸福鄂州，着力推进综合改革示范工作，不断提高城乡人民生活水平，努力建设宜居宜业组群式大城市。

　　我市未来五年经济社会发展的总体目标是：全面完成“十二五”规划和市六次党代会确定的各项任务。经济又好又快发展，到2016年，生产总值达到1000亿元以上，财政总收入达到100亿元以上，人均水平居全省前列；组群式大城市格局基本形成，城乡一体化深入推进，城镇化率达到70%；综合改革示范取得突破，对外开放水平不断提高，自主创新能力显著提升；文化旅游产业加快发展，城市生态优势彰显，“两型”社会建设取得实效；社会管理全面进步，市民幸福指数大幅提升。根据上述总体要求和奋斗目标，今后五年政府工作重点抓好以下五个方面：

　　（一）着力提升产业竞争力，推进跨越发展。一是全力推进工业跨越发展。坚持工业强市，把工业作为产业发展的重中之重，引导资源向工业聚集，加速推进新型工业化。强力实施工业双千亿元产业发展计划，运用高新技术和先进适用技术改造提升冶金、建材、装备制造等传统支柱产业，推动传统支柱产业转型发展。全力支持战略性新兴产业加快发展，大力发展生物医药、电子信息、新能源、新材料等新兴产业。大力实施骨干企业培育工程、高新技术企业发展工程、中小企业成长工程、工业亿元项目招商引资工程、全民创业工程，壮大市场主体，培育一批大企业、大集团，支持一批中型企业成为骨干企业，扶持一批成长性较好的小企业成为规模以上企业，引导一批特色微型企业加快发展，力争形成销售收入过800亿元的产业1个、过200亿元的产业2个、过100亿元的产业4个。二是突破性发展现代服务业。按照建设长江中游区域性物流中转基地、武汉城市圈运动休闲旅游区、金融创新试验区的发展定位，推进服务业跨越发展。加快完善服务业布局，突破性发展生产性服务业，积极发展生活性服务业。以物流、旅游、金融为重点，推进服务业发展提速、比重提高、结构优化。依托武汉新港三江港区发展仓储业、配送中心等生产性物流业，着力构建1000亿元销售规模的物流基地。围绕“一都两湖”发展旅游业，打造区域性湖泊度假旅游目的地。支持市内金融机构创新金融产品，引进股份制商业银行开设分支机构。大力发展中介服务业、商贸流通业、社区服务业、房地产业和建筑业。

　　（二）着力深化综合改革，推进创新发展。坚持以改革推动科学发展、跨越发展，按照“鄂州能突破、全省能推广”的要求，努力创造体制机制新优势。一是围绕重点突破推进改革。努力推进行政管理体制、社会管理体制、城乡一体化、“两型”发展、同城化五大领域改革。坚持试点先行，在行政层级改革、事业单位分类改革、“五个全民”保障体系、社会管理创新、组群式城市发展模式、重工业城市转型发展模式、内陆城市开放模式、“经济人”成长促进机制、农村产权制度改革、投融资体制改革等10个方面率先突破，带动综合改革全面配套推进。二是围绕项目支撑深化改革。按照改革项目化、项目实效化的思路，谋划和建设一批关系综合改革和跨越发展全局的重点项目。集中建设葛店创新成果转化展示工程、三江港现代物流展示工程、大梁子湖生态文明展示工程、鄂城“两型”发展展示工程、长港城乡一体化展示工程、吴都文化产业展示工程等六大综合改革标志性示范工程。三是围绕科技人才保障改革。积极申报全国知识产权示范城市，建立企业技术创新体系、科技创新服务体系、科技投融资体系、财政科技投入稳定增长机制，推进自主创新，加快科技成果转化。实施开放型人才强市战略，推进“千名引硕引博工程”，为推进综改示范、创新发展提供强有力的人才保障。

　　（三）着力加快城乡一体化，推进统筹发展。一是加快建设“全域鄂州”。坚持“全域鄂州、组群发展”的理念，推进主城综合功能区、葛华科技新城、华容临港新城、大梁子湖生态旅游度假区、鄂城转型发展新区等五大功能区的整体规划、整体开发、整体发展，拉开组群式大城市骨架。严格城管执法，开展市容环境综合整治，建立“大城管”体制，提升城市管理水平，营造畅通、靓丽、文明、和谐的城市环境。推进“四城创建”，提升城市形象。二是统筹城乡基础设施建设。加快建设城乡一体的交通、供水、供电、信息、供气、市场、污水处理和垃圾处理网络，实现城乡空间融合、功能互补、基础设施共享。加快农业基础设施建设，改善农村生产条件。继续推进特色镇基础设施建设，完善配套功能，实现特色镇“扩容、提质”。推动农村新社区建设扩面达标，严格按规划和质量标准推进106个农村新社区建设，配套完善基础设施，强化服务功能。三是拓宽农民增收途径。加大政策扶持力度，推进区街经济跨越发展。发展都市农业，壮大水产、畜禽、蔬菜、林果四大支柱产业。落实强农惠农政策，形成稳定的农业投入保障体系。以土地流转提升农业规模化经营水平，大力发展订单农业、家庭农场和股份合作经济。开展农村产权制度改革，完善征地补偿机制，加快建立农民增收的长效机制和利益分享机制，持续增加农民生产性、工资性、财产性和政策性收入。

　　（四）着力建设生态文明，推进绿色发展。一是打造特色文化品牌。突出武昌城的文化特点和梁子湖的生态特色，打造具有鲜明生态特色的鄂州文化品牌。积极申报“中国三国文化之乡”和“吴楚历史文化名城”。以观音阁公园、三国吴都博物馆、西山风景区为载体建设武昌城文化公园，打造具有吴都特色的三国文化旅游品牌。依托大梁子湖生态旅游度假区、葛山文化体育公园建设文化产业园，把文化产业培育成跨越发展的新的支柱产业。实施文化惠民工程，加大体育馆、游泳馆、科技馆等重点工程建设力度，着力构建城乡一体的公共文化服务体系。二是切实加强环境保护。加快建设资源节约型、环境友好型社会，探索跨区域循环经济发展模式。推进环境整治、重工业三废处理与综合利用，从企业“小循环”入手，向行业“中循环”拓展，构建区域“大循环”格局。加强对重点行业、重点企业的监管，进一步降低单位生产总值能耗、化学需氧量排放和二氧化碳排放量。探索湖泊资源生态保护和生态产业发展模式。实施“增绿计划”，着力提高森林覆盖率，建设绿色小区、绿色园区、绿色乡村、绿色廊道，实现绿色生态发展。

　　（五）着力保障和改善民生，推进和谐发展。一是推进全民创业。以创建全省创业型城市为抓手，进一步完善小额担保贷款等政策，鼓励全民创业，以创业带动就业。引导和促进劳动密集型企业和各类服务业加快发展，实施“困难就业人员援助计划”，为群众提供更多的就业岗位。提高中低收入群体收入水平，逐步缩小居民收入差距。二是推进全民教育。优先发展教育，优化城乡教育资源配置，建设城乡群众更满意的优质教育体系。加快发展学前教育，积极创建全省义务教育均衡发展示范市，加快普及高中教育，大力实施素质教育，提高基础教育质量。大力发展职业教育，办好鄂州职教园。加强教师队伍建设，造就一支高素质教师队伍。加快发展继续教育、社区教育和科普教育，构建全民学习、终身教育体系。三是推进全民健康。构建城乡政策均等、与经济发展水平、财政保障能力、群众期待相适应的医疗保障体系。实施城乡统筹、门诊统筹、市级统筹、网络统筹，完善职工医保、居民医保、新农合“三网合一”运行保障机制。深化公立医院改革试点，推进基本药物制度改革，建设基层医疗卫生服务体系，推进基本公共卫生服务均等化。开展全民健身运动，提高全民健康素质。四是推进全民社保。以政策制度全覆盖、制度衔接无障碍、保障需求多样化为目标，加快建立覆盖城乡的社会保障体系。实行城乡居民养老保险一体化、可转换，实现城乡养老、低保、社会救助全覆盖。加快构建新型养老服务体系。五是推进全民安居。实施保障性安居工程，建立住房保障体系，完善保障性住房公平分配制度，缓解中等偏下收入家庭住房困难。六是创新社会管理。深化全省社会管理创新试点工作，建立健全组织、管理、应急、服务、监控“五大体系”，实现由防范控制型向服务管理型转变，形成具有鄂州特色的社会管理模式。提升基层社会自治能力，完善“1+8”社区综合服务中心功能，形成社区服务网络。加快实现社会管理精细化、数字化、动态化。全面推进“六五”普法，深入开展“平安创建”活动，严厉打击刑事犯罪。建立矛盾纠纷大调解格局，畅通信访渠道，依法解决群众合理诉求。完善突发事件应急管理机制，保障人民群众的生命财产安全。

　　三、2012年工作安排

　　2012年是新一届政府的开局之年，也是实施“十二五”规划的攻坚之年，更是推进鄂州跨越发展的关键之年。今年全市经济社会发展的主要预期目标为：地区生产总值增长13.5%；全社会固定资产投资增长25%；地方一般预算收入增长16%；规模以上工业增加值增长20%；社会消费品零售总额增长17%；海关出口增长12%；招商引资总额增长35%，其中实际利用外资增长10%；城镇居民人均可支配收入增长11%；农村居民人均纯收入增长12%；城镇新增就业2万人；城镇登记失业率控制在4.5%以内；人口自然增长率控制在6‰以内；居民消费价格指数与省保持一致；单位生产总值能耗、二氧化碳排放量、主要污染物排放总量完成与省签订的责任目标。今年重点抓好七个方面的工作。

　　（一）以加快发展为首要任务，壮大经济规模。一是招商引资壮规模。大力推进产业招商，吸引相关企业集聚，进一步形成产业集群。继续开展节会招商，充分利用鄂港粤经贸洽谈会、鄂沪经贸洽谈会、厦洽会、中博会等重大节会及其它推介会争取项目合作。注重以商招商，鼓励落户企业增资扩股，积极探索代理招商、委托招商、网上招商等新型招商方式，不断增强招商引资针对性、实效性。二是增加投资壮规模。大力推进项目建设，继续实施“三个一百”工程，确保续建和新开工亿元项目148个、前期推进和招商引资亿元项目120个、争取中省投资项目175个，对项目实行滚动管理，形成开工建设一批、竣工投产一批和策划储备一批的格局。强力推进续建和新开工项目，实行重点项目建设责任制，加强重点项目协调督办，定期召开项目建设协调会，着力解决项目建设相关问题。积极策划一批重大项目，对鄂咸高速、三江港码头、梁子湖第二入江通道等重点项目，明确责任主体，积极争取列入国家、省投资计划。三是扩大出口壮规模。积极开拓国外市场，组织我市外贸企业积极参加广交会、华交会、东盟博览会等各类展会，开拓国际市场，承接出口订单；在招商引资工作中，有针对性地引进外贸出口项目，壮大外贸出口队伍，培植出口后劲。四是激活消费壮规模。大力优化商贸环境，出台鼓励中小商贸企业快速成长的政策，实施“小进限”工程，促进限下商贸流通企业顺利成长，早日进入限上企业行列。强化市场监管，加强对食品的安全监管，密切关注重要商品的价格波动，采取适当措施，保障市场供应。

　　（二）以发展工业为坚实基础，提升产业竞争力。坚持工业强市战略，着力发展实体经济，加大结构调整力度，整体提升鄂州产业综合竞争力。一是加快做大工业规模。强化工业运行调节，加强要素协调。完善中小企业服务体系，着力为企业搭建融资平台，财政安排1000万元用于企业技术改造贷款贴息，100万元奖励金融机构扩大对中小企业贷款。积极培育壮大市场主体，大力实施工业发展五大工程，力争新增销售收入亿元以上工业企业30家、规模以上工业企业30家、高新技术企业6家、小微企业200家，新开工和竣工工业亿元项目60个以上。二是加快调整工业结构。创新工作思路，大力扶持生物医药、新材料等新兴产业发展。完善产业链，重点围绕延伸钢铁上下游产业，加快发展钢铁深加工。抓好“两化”融合试点示范，推进企业信息化工程。加快发展高新技术产业，重点扶持和培育科技型中小企业。创新服务中省企业的工作机制，培育和壮大领军企业。加强企业治乱减负工作。淘汰落后产能。三是加快建设园区平台。促进葛店开发区提档升级，全力争创国家级高新区。加快建设葛店科技企业加速器，完善高新技术产业化基地建设，以生物医药、精细化工、装备制造、电子信息、新能源企业为重点，培育一批地区性、全国性、世界性的知名品牌。切实加大鄂州开发区基础设施建设力度，按照水、电、路、气、通讯一体化开发模式，启动6.5平方公里建设，完善配套基础设施。加快建设三江港园区和梧桐湖新区，完善基础设施，推进项目建设。各开发区按照产业规划的要求，合理定位，错位发展，引导产业向园区集聚，各项政策、要素资源向园区倾斜。四是加快发展现代服务业。落实扶持政策，重点发展旅游文化产业、现代物流业、中介服务业、信息服务业等现代服务业。围绕“一都两湖”，加快推进旅游产品转型升级，积极争创旅游名镇、名村，完善旅游公共保障体系。继续举办梁子湖捕鱼旅游节。加大专业市场建设，改造现有专业市场，加快新型专业市场建设，拓宽消费领域，改善消费环境。加快发展金融保险业，创新金融产品，支持顾地、大通等企业上市。发展社区商业、物业、家政等服务业。

　　（三）以改革创新为强大动力，推进综改示范工作。重点推进五项改革。一是推进行政层级改革。重点推进功能区改革试点，在条件成熟的试点地区搭建“一级政府三级管理”组织架构,着力理顺市区两级政府职能，以推进政府公共服务、基层民主自治和社会综合治理有机融合、良性互动为重点，完善新社区管理体制和运行机制，为在更大范围内推进行政层级改革积累经验、创造条件。二是推进行政审批制度改革。进一步畅通行政审批“绿色通道”，坚持一站式审批、一条龙服务、一个窗口收费，优化服务流程、提升服务质量，在市行政服务中心设立“绿色通道”首受窗口和审批窗口，实行首问负责制，并联审批、封闭运行、超时默许、统一收费、失责追究。对所有进入市、区行政服务中心的行政审批事项全部纳入行政审批电子监察系统。三是推进农村产权制度改革。重点推进农村集体土地、宅基地、房屋确权改革，进行农村产权资本化试点，建立农村综合产权交易体系。选择1-2个试点区域，由市或功能区组建的现代农业开发公司、城镇建设投资公司主导或引导农村综合产权交易。加快推进农村信用社改制为农村商业银行。探索建立以农村土地承包经营权、养殖水域承包经营权和林权等资产为抵押、质押担保的融资体系。建立农村金融担保体系和农村产权金融风险分担及补偿机制。四是推进“两型”社会改革。组织研究建立“两型”社会生产标准体系，形成“两型”产业分类标准、“两型”企业评价标准。建立“两型”社会生活标准体系，形成“两型”建筑标准、出行标准、消费标准。集中力量打造2-5个“两型”企业、循环经济园区和绿色小城镇、低碳新社区样板。积极开展梁子湖国家湖泊生态环境保护试点，大力推进重点流域水污染防治。加快推进重点减排工程建设，强化对重点污染源污染防治设施的运行监管。五是推进基层社会管理创新。在新社区建立政府公共服务平台，大力培育和发展社区服务组织、中介组织和专业性社会工作机构，创建一批以社区党组织为核心、社区自治组织为主体、政府基层公共服务平台为依托、社区集体经济组织为补充、社区居民广泛参与的基层社会管理新样板。

　　（四）以城乡一体化为战略重点，统筹推进“三农”工作。一是大力建设特色镇和新社区。市区镇三级继续筹资6000万元，重点支持特色镇完善功能配套，着力打造工业重镇、商贸强镇、旅游文化名镇，积极推进镇级市试点。创新城镇管理体制机制，加快培育小城镇主导产业，提升城镇承载能力和辐射功能。建好农村新社区，积极引导农民转变生产生活方式。加大体制机制创新，积极探索农村治理新模式。结合农村新社区建设，推进农村环境连片整治。二是大力改善农业生产条件。统筹城乡水利、交通、信息、市场、供排水、供电、供气等基础设施建设，强力推进城市基础设施向农村延伸。扎实开展“万名干部进万村挖万塘”活动，确保取得实效。加强农田水利基本建设，继续做好长港河整治工程、南迹湖泵站更新改造、磨刀矶节制闸整险加固和中小河流、水库、湖泊整治等工程建设。三是大力发展都市农业。提高农业科技水平，增强农业综合生产能力，发展四大支柱产业，推进农业产业化，围绕“四个一批”工程培育壮大一批重点龙头企业和一批知名品牌。加快培育和发展农业专业合作组织，实施农业产业化龙头工程，力争新增销售收入过1亿元企业2-3家。加快发展休闲农业和观光农业，培育农业新的经济增长点。做好防汛抗旱和自然灾害防治工作，确保农业增效、农民增收。

　　（五）以建设组群式大城市为发展目标，加快城市建设步伐。一是不断增强城市功能。计划投资15亿元建设主城区基础设施，重点打造精致城市的载体和平台。加快建设吴楚大道、鄂州大道（南北中轴线）、鄂东大道、葛山大道、凤凰大桥、三国吴都风光带三期、洋澜湖生态公园二期、洋澜湖环湖截污工程、五丈港疏浚工程、火车站广场等十大重点工程，续建和新建汇贤路、沿江大道东段、月河桥等道路桥梁工程，启动吴都休闲景观带等大型园林景观工程。新增城市绿地18公顷，城市污水处理率达到82%。配套推进供排水、燃气管道建设和城市道路及景观照明节能改造，加快推进公共停车设施规划建设，实施老城区雨污分流改造。二是提升鄂州交通枢纽地位。争取汉鄂高速7月建成通车。加快鄂咸高速前期工作，力争早日动工。全力推进武汉新港三江港区建设，加快五丈港区二期工程、三和管桩综合码头、超凡物流码头等建设进度。完成东沟大桥建设任务。三是努力提升城市管理水平。加大城市管理综合整治力度，突出市容环境整治，从严控制和查处违法建设，基本实现主城区主干道无出店占道经营、市容整洁有序。全面提升环境卫生质量，主城区一级垃圾清运实现机械化。加强市政设施管养，市政设施综合完好率达到98%。

　　（六）以保障和改善民生为根本目的，提高市民幸福指数。一是努力改善民生。积极促进和扩大就业，完善公共就业服务体系，扩大小额担保贷款，推进全民创业，争创全省创业型城市。以城乡全覆盖为目标，推进城乡社会保障体系建设。完善养老保险制度体系，继续加强城乡居民社会养老保险工作。推进事业单位养老保险制度改革。完善城乡一体的医疗保险政策、管理和服务体系。完善工伤保险体系，加强失业、生育保险工作。强化社保基金监管。继续提高低收入群体保障水平。健全城乡低保制度，完善医疗救助政策。扎实开展“和谐社区”创建活动，大力发展社区社会化服务。落实优待抚恤政策。提高城乡退役义务兵安置水平。发展社会化养老服务，兴办公益性养老机构。提高残疾人康复、托养、就业水平。二是大力发展社会事业。加快推进义务教育学校布局调整和标准化建设，全面开展义务教育均衡发展改革试验。抓紧实验小学建设，确保秋季开学。加快发展学前教育，新建、改扩建一批城乡幼儿园。深入推进新课程改革，促进普通高中特色发展，全面推进素质教育，提高办学水平和教育质量。建立完善中小学教师补充、培训、交流机制，提高教师专业化水平。大力发展职业教育，加快鄂州职教园建设。加快发展文化体育事业。推进文化体制改革，完成国有文艺院团改制。全面完成新博物馆建设并对外开放，扎实推进文化体育产业园等重点项目建设。继续实施文化惠民工程，统筹城乡广电事业发展。深入推进医药卫生体制改革，完善基本医疗卫生服务体系。统筹推进公立医院改革试点工作。加大传染病防治力度，为城乡居民免费提供基本公共卫生均等化服务。完善人口计生公共服务，稳定低生育水平。三是全力维护社会稳定。加强社会管理创新，大力推广运用网格化、信息化管理模式和技术手段，加快实现社会管理精细化、数字化、动态化。高度重视安全生产和应急管理工作，落实企业安全生产主体责任，加强群众安全知识宣传教育。强化安全生产监督管理，预防和减少各类事故。加强工商行政管理、质量技术监督和食品、药品、烟草市场管理，保护消费者权益。开展风险隐患排查治理，加强突发公共事件的信息报告和预警工作，提高应对突发公共事件的能力。四是认真做好相关工作。依法管理民族宗教事务。做好外事侨务工作。重视人口老龄化问题。关心和支持青年、妇女、儿童事业发展。推进新一轮援疆工作，做好对口支援民族县市工作。认真做好物价、统计、公积金管理、邮政、石油石化、防震减灾、档案、方志、人防、消防、扶贫开发、农机、气象和国防教育、民兵建设等工作。五是坚持办好“十件实事”。多方筹资10.76亿元，办好加强就业和再就业工作、提高社会保障水平、促进教育事业发展、提升医疗卫生服务水平、提高农民生活质量、优化农民生活环境、改善农村生产条件、改善城乡居民生活环境、改善市民出行条件、加强社会管理创新等“十件实事”。

　　（七）以优化发展环境为重要抓手，建设人民满意的政府。一是推进依法行政，建设法治政府。加强行政决策程序建设，健全重大行政决策规则，完善行政决策风险评估机制，推进行政决策科学化、民主化、法治化。深入推进行政执法体制改革，规范行政执法行为，提高基层依法行政能力。自觉接受人大及其常委会的法律监督和工作监督、政协的民主监督，广泛听取民主党派、工商联和工青妇等人民团体以及社会各界人士的意见。推进政府信息公开，完善新闻发布制度，提高政府工作透明度。加强行政复议委员会建设，完善相对集中行政复议权工作制度，发挥行政复议化解行政争议主渠道作用，保障相对人合法权益。二是转变政府职能，建设高效政府。完善市政府运作机制，进一步梳理行政职权，加快推进政企分开、政资分开、政事分开、政府与市场中介组织分开，切实转变政府管理经济方式，加强公共服务和社会管理职能。健全行政问责制，切实提高政府公信力。继续深化“治庸问责”活动，促进行政服务提速增效。三是提高政府执行力，建设务实政府。继续加强政府执行力建设，切实转变机关作风，在全市各级政府部门大力推广“马上就办”的办事理念和机关文化，定下来的事雷厉风行，看准了的事一抓到底。敢于突破、敢于负责、勇于担当，于困境中找到出路、于困难中找到办法、于可能中办成事情。大力推进政府绩效管理，以服务群众、服务基层、服务发展的实绩检验政府行政效能。强化监督检查，保证政令畅通。四是加强廉政建设，建设廉洁政府。认真贯彻执行廉政准则，深入开展反腐倡廉教育，加强对领导干部个人重大事项报告制度执行情况的监督检查，加强对配偶子女均已移居国（境）外的公职人员的管理。加强行政监察和审计监督，规范公共资源交易和招投标行为，抓好工程建设等重点领域和关键环节的反腐倡廉制度建设，推进廉政风险预警防控工作，完善惩治和预防腐败体系。坚决查办违纪违法案件
